# An overview of combined D-2- and L-2-hydroxyglutaric aciduria: functional analysis of CIC variants

**DOI:** 10.1007/s10545-017-0106-7

**Published:** 2017-12-13

**Authors:** Ana Pop, Monique Williams, Eduard A. Struys, Magnus Monné, Erwin E. W. Jansen, Anna De Grassi, Warsha A. Kanhai, Pasquale Scarcia, Matilde R. Fernandez Ojeda, Vito Porcelli, Silvy J. M. van Dooren, Pascal Lennertz, Benjamin Nota, Jose E. Abdenur, David Coman, Anibh Martin Das, Areeg El-Gharbawy, Jean-Marc Nuoffer, Branka Polic, René Santer, Natalie Weinhold, Britton Zuccarelli, Ferdinando Palmieri, Luigi Palmieri, Gajja S. Salomons

**Affiliations:** 1 Metabolic Laboratory, Department of Clinical Chemistry, Amsterdam Neuroscience, VU Medical Center Metabolic Unit PK 1X009, Postbus 7057, 1007 MB Amsterdam, The Netherlands; 2 Department of Biosciences, Biotechnologies and Biopharmaceutics, University of Bari, Bari, Italy; 3 Department of Sciences, University of Basilicata, Potenza, Italy; 4 Division of Metabolic Disorders, CHOC Children’s, Orange, CA USA; 5 Department of Pediatrics, University of California at Irvine, Irvine, CA USA; 6 Department of Metabolic Medicine, Lady Cilento Children’s Hospital, Brisbane, Australia; 7 School of Medicine, University of Queensland Brisbane, Griffith University Gold Coast, Gold Coast, Australia; 8 Clinic for Pediatric Kidney-, Liver- and Metabolic Diseases, Hannover Medical School, Hannover, Germany; 9 Department of Pediatrics and Division of Medical Genetics, University of Pittsburgh School of Medicine, Pittsburgh, PA USA; 10 Division of Pediatric Endocrinology, Diabetology and Metabolism and University Institute of Clinical Chemistry, Inselspital, University Hospital, University of Bern, Bern, Switzerland; 11 Department of Pediatrics, PICU, University Hospital Centre, Split, Croatia; 12 Department of Pediatrics, University Medical Center Hamburg Eppendorf, Hamburg, Germany; 13 Sozialpädiatrisches Zentrum, Charité Universitätsmedizin Berlin, Berlin, Germany; 14 The University of Kansas School of Medicine Salina Campus, Salina, USA; 15 Institute of Biomembranes, Bioenergetics and Molecular Biotechnology, Consiglio Nazionale delle Ricerche, Bari, Italy

**Keywords:** Mitochondrial citrate carrier, SLC25A1, Structure-function correlations, Residue specific score, Structural homology, Krebs cycle intermediates

## Abstract

Combined D-2- and L-2-hydroxyglutaric aciduria (D/L-2-HGA) is a devastating neurometabolic disorder, usually lethal in the first years of life. Autosomal recessive mutations in the *SLC25A1* gene, which encodes the mitochondrial citrate carrier (CIC), were previously detected in patients affected with combined D/L-2-HGA. We showed that transfection of deficient fibroblasts with wild-type *SLC25A1* restored citrate efflux and decreased intracellular 2-hydroxyglutarate levels, confirming that deficient CIC is the cause of D/L-2-HGA. We developed and implemented a functional assay and applied it to all 17 missense variants detected in a total of 26 CIC-deficient patients, including eight novel cases, showing reduced activities of varying degrees. In addition, we analyzed the importance of residues affected by these missense variants using our existing scoring system. This allowed not only a clinical and biochemical overview of the D/L-2-HGA patients but also phenotype–genotype correlation studies.

**Electronic supplementary material:**

The online version of this article (10.1007/s10545-017-0106-7) contains supplementary material, which is available to authorized users.

## Introduction

Combined D-2- and L-2-hydroxyglutaric aciduria (D/L-2-HGA; OMIM #615182) was described as the third variant of 2-HGA, clinically manifesting a severe phenotype with neonatal encephalopathy, respiratory insufficiency, developmental delay, hypotonia, and early death (Muntau et al. 2000). This condition is biochemically characterized by accumulation of both enantiomers of 2-hydroxyglutaric acid (2-HG), with a more pronounced D-2-HG increase in bodily fluids. Recessive mutations in the gene *SLC25A1* (NM_005984) are the underlying genetic cause of D/L-2-HGA (Edvardson et al. 2013; Nota et al. 2013). *SLC25A1*, located on chromosome 22q11, encodes for the mitochondrial citrate carrier SLC25A1 (CIC). This protein belongs to the SLC25 family of mitochondrial carriers (MC), which are mainly localized in the inner mitochondrial membrane and are responsible for the trafficking of a variety of metabolites (Palmieri 2013; Palmieri and Monné 2016). All SLC25 members are characterized by a tripartite structure and topologically by six transmembrane α-helices (Klingenberg 1989; Kaplan et al. 1993; Palmieri 2013).

The CIC is a protein of 311 amino acids that mediates the exchange of mitochondrial citrate/isocitrate for cytosolic malate (Palmieri 2004). Citrate, an important component of energy metabolism, is mainly produced in the mitochondria end—to a much lesser extent—in the cytoplasm by reductive carboxylation (Jiang et al. 2016). Citrate is also taken up from the blood stream (mainly) via the SLC13A5 sodium-dependent plasma membrane citrate transporter (Inoue et al. 2002; Bhutia et al. 2017). The mitochondrial citrate is either used as an intermediate of the Krebs cycle or transported outside the mitochondria by the CIC, where it plays important roles in fatty acid and sterol synthesis, regulation of glycolysis, histone acetylation, and other physiopathological processes (Mycielska et al. 2009; Iacobazzi and Infantino 2014).

When the CIC function is impaired, it is presumed that mitochondrial citrate accumulates, and as a result, cytosolic citrate concentration decreases (Nota et al. 2013). Studies of primary deficient fibroblasts grown in [U-^13^C_6_] glucose-enriched medium showed lower [^13^C_2_] citrate levels in culture medium than in controls (Nota et al. 2013). Also, individuals with mitochondrial citrate deficiency have D/L-2-HGA and, as a group, lower urinary levels of citrate (and, to some extent, also isocitrate), and higher urinary levels of Krebs cycle intermediates downstream of citrate (α-ketoglutarate, succinate, fumarate, and malate) compared with controls (Nota et al. 2013; our study, Supplementary Table 1).

Fourteen genetically confirmed cases with combined D-2- and L-2- HGA have been reported with varying, usually severe, clinical presentation (Muntau et al. 2000; Read et al. 2005; Edvardson et al. 2013; Nota et al. 2013; Mühlhausen et al. 2014; Prasun et al. 2015; Smith et al. 2016). Two additional cases (a sibling pair) associated with SLC25A1 deficiency presented a milder clinical phenotype with impaired neuromuscular transmission and no HGA (Chaouch et al. 2014).

To date, 16 mutations throughout the *SLC25A1* gene have been reported (Edvardson et al. 2013; Nota et al. 2013; Prasun et al. 2015; Smith et al. 2016). Most (75%) described mutations are missense. Analysis of missense variants by commonly used software prediction tools is a challenge. We applied the scoring system for SLC25 members (Pierri et al. 2014) to characterize the importance of the involved individual amino acids. In addition, we developed and implemented a functional assay for analysis of the SLC25A1 missense variants, followed by genotype–phenotype studies.

## Materials and methods

### Patients, clinical and biochemical data

Inclusion criteria for this study were the presence of *SLC25A1* variants and combined D/L-2-HGA; 26 individuals were evaluated. Clinical data was collected from referring physicians using questionnaires. For previously published case reports (Muntau et al. 2000; Read et al. 2005; Edvardson et al. 2013; Chaouch et al. 2014; Mühlhausen et al. 2014; Prasun et al. 2015; Smith et al. 2016), data was completed by two of our authors (MW, AP) based on published information. No clinical data could be obtained for patient nos. 2 and 26 (sibling of patient no. 3). The D/L-2-HGA biochemical diagnosis was, in most cases (*N* = 22), established by our laboratory using liquid chromatography tandem mass spectroscopy (LC-MS/MS) measurements of D-2-HG and L-2-HG in urine, as previously described (Struys et al. 2004), or the information was collected via questionnaires (*N* = 1) or from literature (*N* = 3; two of three were reported not to have increased urinary D-2-HG and L-2-HG) (Chaouch et al. 2014).

### Genetic testing

For seven of the unpublished D/L-2-HGA-affected individuals, all exons and adjacent splice sites of the *SLC25A1* coding region were amplified by polymerase chain reaction (PCR), as previously described (Nota et al. 2013). Sequencing analysis was performed using an ABI 3130xl genetic analyzer (Applied Biosystems, Nieuwekerk a/d Ijssel, NL), and data was interpreted using Mutation Surveyor (Softgenetics, PA, USA). Whole-exome sequencing, followed by direct Sanger sequencing, resulted in the genetic diagnosis in another affected individual.

### Construction of the expression vector and site-directed mutagenesis to introduce missense variants

The coding sequence of the *SLC25A1* gene was recloned from pCMV6-AC-GFP (Origene, Rockville, MD, USA) into the pEGFP-N1 vector (Clontech). Subsequently, the enhanced green fluorescent protein (EGFP) was removed from the vector, as it interfered with protein function. For each of the 17 missense mutations included in this study, recombinant plasmids were generated by site-directed mutagenesis, as previously described (Betsalel et al. 2012). Successful mutagenesis and absence of PCR artifacts was confirmed by full-length sequencing of the *SLC25A1* coding sequence.

### Restoration of the primary defect and overexpression studies

*SLC25A1* ^−/−^ fibroblasts from patient 9, homozygous for c.18_24dup; p.Ala9Profs*82 mutation, were transfected with wild-type *SLC25A1* (wt), empty vector or mock transfected, by electroporation using 4D–Nucleofector™ system and P2 primary cell kit (Lonza, Cologne, Germany), following the manufacturer’s guidelines. Thereafter, the 17 *SLC25A1* constructs were transiently transfected in *SLC25A1* ^−/−^ fibroblasts using 1.5 million cells per condition. The pEGFP-N1 was cotransfected with the CIC-expressing vectors (with or without the introduced variants) in a ratio of 1 to 100. All experiments were performed in triplicate.

### Functional studies

Twenty-four hours after transfection, cells were incubated for 48 h with Dulbecco’s modified Eagles medium (DMEM) enriched with [U-^13^C_6_] glucose. Thereafter, culture media and cell pellets were collected and stored at −20 °C and −80 °C, respectively. To assess restoration of the CIC function in wild-type transfectants and activity of mutated proteins, [^13^C_2_] citrate levels in cell culture media of transfected fibroblasts were used, as previously described (Nota et al. 2013). The percentage of residual CIC activity is expressed to the activities of wild-type transfectants, which were arbitrarily set at 100% in each experiment.

### Confirmation of successful transfection by Western blotting

Transfected cells were subjected to sodium dodecyl sulfate polyacrylamide gel electrophoresis (SDS-PAGE) and Western blot analysis. First, cells were lysed in urea lysis buffer (8 M urea, 100 mM NaCl, 10 mM Tris-HCl, pH 8.0) and sheared through an insulin syringe needle for DNA disruption. Protein content was determined using a bicinchoninic acid protein assay (Sigma-Aldrich, St Louis, MO, USA). Proteins (17 μg) were size-separated in a 12% NuPAGE® Bis-Tris precast gel (Invitrogen, Carlsbad, CA, USA) and transferred to a polyvinylidene fluoride (PVDF) membrane using the iBlot® Dry Blotting System (Invitrogen). Immunodetection of the SLC25A1 protein was carried out using rabbit polyclonal anti-SLC25A1 primary antibody (Proteintech, 15,235–1-AP), polyclonal goat anti-rabbit immunoglobulins/horseradish peroxidase (HRP) secondary antibody (Dako, P 0448) and enhanced chemiluminescent substrates (Lumi-Light plus Western blotting substrate; Roche Applied Science, Indianapolis, IN, USA). Images were acquired with the ChemiDoc MP imager (Bio-Rad Laboratories) and analyzed using Image Lab software. Actin was used as an internal loading control.

### Residue-specific scores of mutated residues

Using the scoring systems developed by Pierri et al. 2014, functional and/or structural importance of mutated residues was evaluated. The residue-specific score (RS) estimates the strength of the evolutionary selection on each amino acid residue of an individual mitochondrial carrier. Considering all 216 residues previously scored for SLC25A1 (Pierri et al. 2014), a residue with an RS > 4.68 is above the median value and therefore considered functionally and/or structurally important in the human CIC. A transversal score (TS) was calculated by averaging the 53 residue-specific scores corresponding to equivalent positions identified by the multialignment of the 53 members of the human mitochondrial carrier protein family (MCF), using the *Bos taurus* protein BtAAC1 structure as reference. A residue with a TS > 3.79 is above the median value and considered functionally and/or structurally important in the common MCF structure and transport mechanism.

### Structural homology model of the human CIC

The homology model of human CIC (residues 23–299) was made with MODELER (Fiser and Sali 2003) based on the X-ray structure of the bovine adenosine diphosphate/adenosine triphosphate (ADP/ATP) carrier (Pebay-Peyroula et al. 2003).

## Results

### *SLC25A1* gene sequencing results

**Fig. 1:**
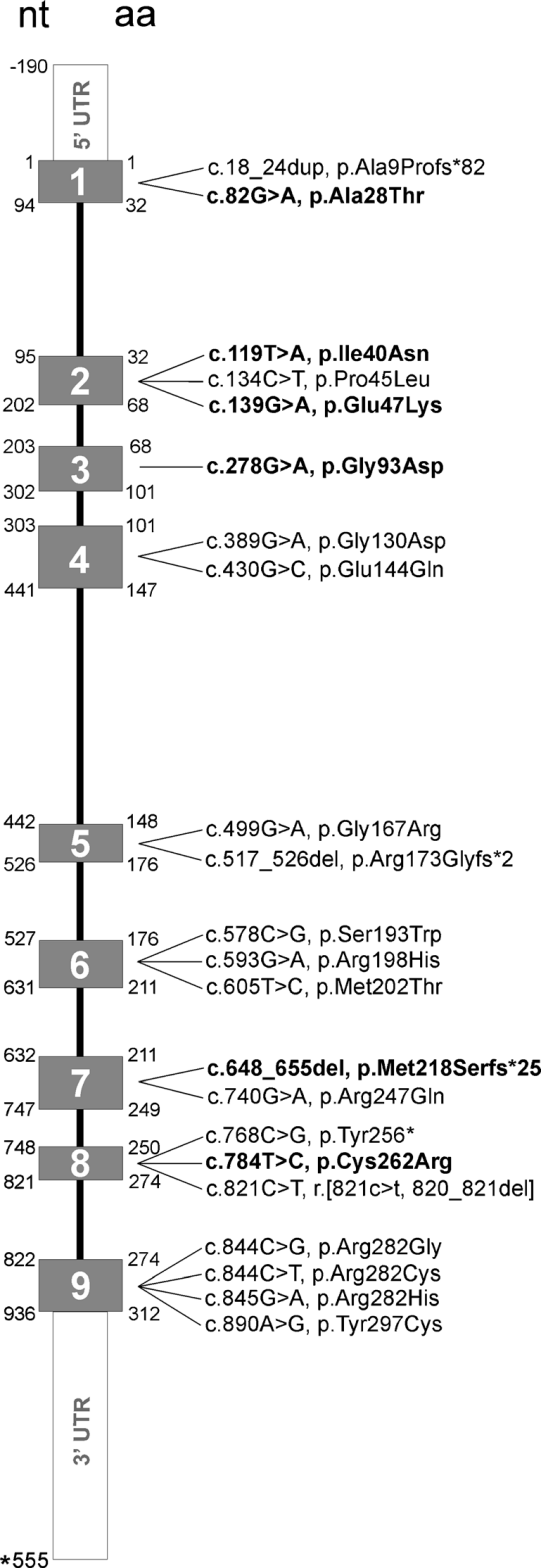
*SLC25A1* gene showing distribution of all currently known mutations. These mutations are part of the mutations database LOVD (http://www.lovd.nl/slc25a1). Novel mutations described in this study are represented in bold

An overview of all 22 mutations found in the *SLC25A1* gene, including six novel, is given in Fig. 1. Most of these mutations are private, and only seven mutations were found in more than one patient. The most frequent mutation is p.Ala9Profs*82, detected in five patients (four apparently unrelated families).

### Presentation and clinical features

Presenting symptoms are summarized in Table 1. Dysmorphic features seen more than once are prominent forehead–frontal bossing in four patients, bitemporal hypoplasia–midface hypoplasia in three, hypertelorism in four, and down-slanting eyes in three. A low or flat nasal bridge was seen in four, low-set or rotated ears in three, abnormal thumbs in two, micrognathia in three, and retrognathia in two. Head circumference was normal in seven; six patients were microcephalic and one macrocephalic. Weight was normal in ten, and four patients had growth retardation. Delayed motor milestones were reported in 16 patients, with no development at all in two patients. Motor function evaluation was not determined or given in seven cases. Cognitive development was impaired in 13 patients, and no development was observed in three. No specific tests were performed to evaluate cognition. Other specific abnormalities are optic atrophy, seen in patient 1, a feature described previously by Edvardson et al., in patient 22.

**Table 1:** Overview of genotype and phenotype of 26 mitochondrial citrate carrier (CIC)-deficient patients

Patients^a^	Presentation (days)^b^	First symptoms				Features at follow-up				Allele 1			Allele 2			Patient groups^f^	Clinical severity
		Respiratory problems^c^	Hypotonia	Seizures	Encephalopathy	Respiratory problems^c^	G-tube	Dysmorfism	Age (months)^d^	Nucleotide change	Deduced effect	Residual activity^e^	Nucleotide change	Deduced effect	Residual activity^e^		
1^g^	10	Yes-A	Yes	Yes	–	Yes-V	–	Yes	21	**c.578C > G**	**p.Ser193Trp**	31% (8)	**c.578C > G**	**p.Ser193Trp**	31% (8)	3	mild
2	–	–	–	–	–	–	–	–	–	**c.844C > G**	**p. Arg282Gly**	7% (9)	**c.844C > G**	**p. Arg282Gly**	7% (9)	1	–
3^g,h,i,j^	IUGR**	–	Yes	–	–	Yes-A	–	Yes	† 4	c.844C > T	p.Arg282Cys	5% (5)	c.844C > T	p.Arg282Cys	5% (5)	1	severe
4^g,h^	–	–	Yes	–	–	Yes-A	–	Yes	† 61	c.821C > T	r.[821c > t, 820_821del]	*85% (9)	c.821C > T	r.[821c > t, 820_821del]	*85% (9)	3	mild
5	1	–	–	–	Severe	Yes-insufficiency	–	Yes	† < 24	c.18_24dup	p.Ala9Profs*82	–	c.499G > A	p.Gly167Arg	49% (4)	2	severe
6^k^	< 7	Yes-V	–	Yes	–	Yes-V	Yes	Yes	† 2	c.18_24dup	p.Ala9Profs*82	–	c.134C > T	p.Pro45Leu	23% (20)	1	severe
7	1	–	Yes	–	–	Yes-A	–	–	† 1	c.18_24dup	p.Ala9Profs*82	–	c.768C > G	p.Tyr256*	9% (8)	1	severe
8	1	Yes-A	–	–	Severe	Yes-A	–	Yes	† 1	c.430G > C	p.Glu144Gln	1% (3)	c.430G > C	p.Glu144Gln	1% (3)	1	severe
9	< 7	Yes-A	Yes	–	–	Yes-V	Yes	Yes	† 11	**c.18_24dup**	p.Ala9Profs*82	–	**c.18_24dup**	p.Ala9Profs*82	–	1	severe
10^i^	90	–	Yes	–	–	Yes-A	Yes-intermittent	–	† 132	**c.605 T > C**	**p.Met202Thr**	66% (23)	**c.890A > G**	**p.Tyr297Cys**	30% (14)	3	mild
11	–	–		–	–	Yes-insufficiency	–	–	† 30	c.821C > T	r.[821c > t, 820_821del]	*85% (9)	c.821C > T	r.[821c > t, 820_821del]	*85% (9)	3	mild
12^g^	IUGR**	Yes-A	Yes	–	–	Yes-A	No	–	60	**c.517_526del**	p.Arg173Glyfs*2	–	**c.821C > T**	**r.[821c > t, 820_821del]**	*85% (9)	2	mild
13	35	–	Yes	–	Mild	Yes-V	Yes	–	† 5	c.18_24dup	p.Ala9Profs*82	–	c.18_24dup	p.Ala9Profs*82	–	1	severe
14	hydrocephalus**	Yes-A	Yes	Yes	Severe	Yes-V	No (nasogastric)	Yes	† 11	**c.517_526del**	p.Arg173Glyfs*2	–	**c.517_526del**	p.Arg173Glyfs*2	–	1	severe
15	9	Yes	–	–	–	Yes-V-intermittent	Yes	Yes	21	c.784 T > C	p.Cys262Arg	48% (8)	c.784 T > C	p.Cys262Arg	48% (8)	3	severe
16	1	Yes-V	–	–	–	Yes-V	Yes	–	† 1	c.139G > A	p.Glu47Lys	8% (10)	c.139G > A	p.Glu47Lys	8% (10)	1	severe
17	2	–	–	Yes	–	Yes-A	Yes	Yes	† 5	c.278G > A	p.Gly93Asp	7% (1)	c.278G > A	p.Gly93Asp	7% (1)	1	severe
18	1	Yes-A	–	Yes	–	Yes-V	–	Yes	50	c.593G > A	p.Arg198His	11% (11)	c.593G > A	p.Arg198His	11% (11)	1	severe
19	ventriculomegaly**	Yes-A	–	Yes	–	Yes-V	Yes	–	23	c.119 T > A	p.Ile40Asn	34% (13)	c.648_655del	p.Met218Serfs*25	–	2	severe
20^i^	60	Yes	Yes	–	–	Yes-A-intermittent	Yes >7 months	–	38	c.82G > A	p.Ala28Thr	71% (14)	c.578C > G	p.Ser193Trp	31% (8)	3	mild
21	90	Yes-A	Yes	–	–	Yes-V-intermittent	No	–	60	c.605 T > C	p. Met202Thr	66% (23)	c.844C > T	p.Arg282Cys	5% (5)	2	mild
22	10	Yes-A	Yes	–	Mild	Yes-V	Yes	–	18	c.845G > A	p. Arg282His	1% (2)	c.389G > A	p.Gly130Asp	16% (4)	1	severe
23^k^	1	Yes-V	–	Yes	Severe	Yes-V	–	Yes	† 0.75	c.18_24dup	p.Ala9Profs*82	–	c.134C > T	p.Pro45Leu	23% (20)	1	severe
24^l^	*< 2 years*	–	Yes-periferal	–	–	No	No	–	33 years	c.740G > A	p. Arg247Gln	52% (9)	c.740G > A	p. Arg247Gln	52% (9)	3	mild
25^l^	*< 2 years*	–	Yes-periferal	–	–	No	No	–	19 years	c.740G > A	p. Arg247Gln	52% (9)	c.740G > A	p. Arg247Gln	52% (9)	3	mild
26^j^	–	–	–	–	–	–	–	–	–	c.844C > T	p.Arg282Cys	5% (5)	c.844C > T	p.Arg282Cys	5% (5)	1	–

Bold indicates compound heterozygosity or homozygosity confirmed in our laboratory by DNA sequence analysis of the parents

*Citrate efflux of the p.Ala274Val allele was tested and showed 85% of residual activity. It should be noted that in fibroblasts, this transcript was only faintly detected in Western blot, and it is unknown whether its expression is different in other tissues. However, 57% of residual activity was detected in primary deficient fibroblasts of patient 4, suggesting that this transcript and/or the frameshift transcript have relatively high residual activity (see section: Results and Discussion)

** Prenatal

^a^Patients 1–12 are briefly described by Nota et al. 2013; patient 6 by Prasun et al. 2015; patient 12 by Mühlhausen et al. 2014; patient 18 by Smith et al. 2016; patient 22 by Edvardson et al. 2013; patients 24–25 by Chaouch et al. 2014

^b^ *IUGR* intrauterine growth restriction

^c^ *V* ventilated,* A* apnea

^d^Age at questionnaire completion/in publication

^e^Percentage of activity is the mean of three independent experiments compared with wild-type transfectants, which are set as 100% (standard deviation). The overall trend of residual activities was comparable in each experiment. Western blotting was performed to confirm construct validity by showing CIC protein expression (Supplementary Fig. 1)

^f^Patient groups as discussed in Results section

^g^Patients also had no eye contact as first symptoms

^h^Patients also had no mimic as first symptoms

^i^Patients also had ptosis as first symptoms

^j^Patients 3 and 26 are siblings

^k^Patients 6 and 23 are siblings

^l^Patients 24 and 25 are siblings

At follow-up, epilepsy becomes a more prominent feature. Seizures were seen clinically or on electroencephalogram (EEG) in 20 patients, apnea was reported as seizure in three cases. Four patients showed no seizures, but this was on the grounds of a normal EEG during apneic attacks. Radiographic information from magnetic resonance imaging (MRI), computed tomography (CT scan), or brain ultrasound (US) investigations showed brain atrophy and/or ventriculomegaly in 14 of 20 patients and corpus callosum hypoplasia or dysplasia in 12 of 20. Germinolytic cysts or germinal matrix cysts were seen in two patients. Bilateral frontal lobe cysts, evidence of prenatal intraventricular bleeding, and cerebellar hypoplasia and gliotic hyperintensity of frontal white matter were seen separately in individual cases. Brain MRI of patient 24 was normal (Chaouch et al. 2014). Initial laboratory findings revealed lactic acidosis in 14 of 17 cases. Urinary levels of Krebs metabolites of seven of eight unpublished patients are shown in Supplementary Table 1.

### Restoration of CIC function by overexpression of wild-type* SLC25A1*

**Fig. 2:**
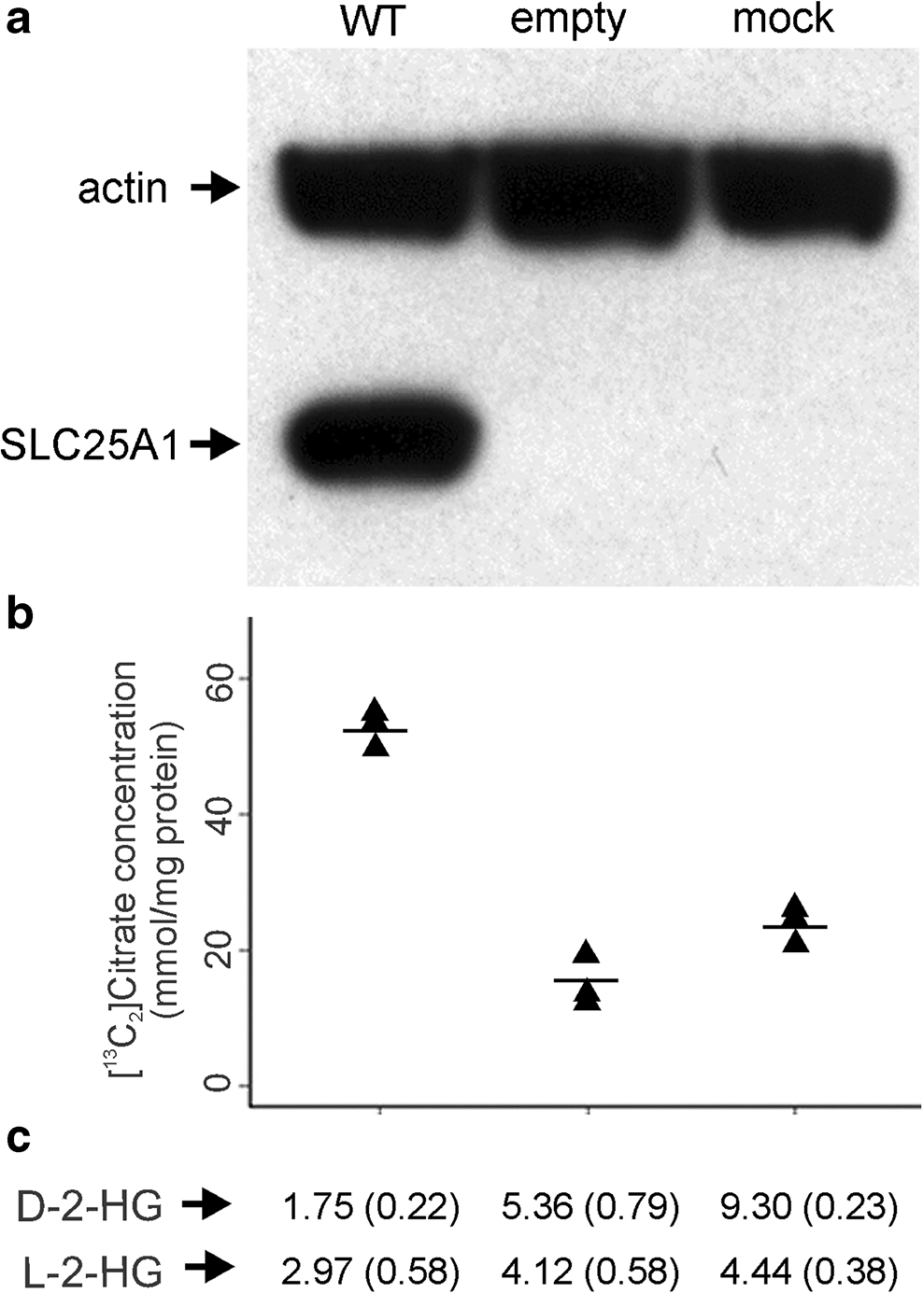
Restoration of mitochondrial citrate carrier (CIC) function by overexpression of wild-type SLC25A1 in *SLC25A1* ^*−/−*^ fibroblasts. **a** Detection of overexpressed protein by Western blotting. **b** [^13^C_2_] citrate levels measured in culture medium by liquid chromatography tandem mass spectroscopy (LC-MS/MS) show increased levels in wild-type transfectants compared with empty and mock transfectants.* Horizontal lines* indicate the group sample mean (*n* = 3). **c** Mean D-2-hydroxyglutaric acid (HG) and L-2-HG intracellular levels (standard deviation). The abundance of formed L-2-HG and D-2-HG isotopomers were so low that quantification was hampered; however, unlabeled L-2-HG and D-2-HG were quantifiable, and these values are depicted here

Transfection of *SLC25A1* ^−/−^ fibroblasts with wild-type *SLC25A1* not only restored SLC25A1 levels, as detected by immunoblotting (Fig. 2a), but also resulted in the expected increase of citrate efflux (by > 3-fold compared with cells transfected with an empty vector) (Fig. 2b), accompanied by decreased intracellular D-2-HG and L-2-HG levels (Fig. 2c).

### Functional studies: genotype–phenotype correlations

Residual activities of studied missense variants varied from almost 0 to 85% of that of wild-type transfectants. Patients with a severe clinical outcome—death before 1 year and/or intensive medical care—had missense variants with residual activities up to 23% and/or severe truncating mutations (i.e., nonsense or frameshift), considered to result in no CIC activity. Therefore, by using the 25% residual activity as cutoff value, we divided the D/L-2-HGA patient cohort into three groups: (1) patients with two severely affected CIC alleles (truncating or missense mutations with < 25% residual activity); (2) patients compound heterozygous for a severely and a mildly affected allele; (3) patients with two relatively mildly affected CIC alleles (with residual activity > 25%).

In group 1, we included four patients carrying two truncating mutations and eight (nos. 3, 6, 8, 16, 17, 18, 22, and 23) with missense variants showing severe impairment of the CIC function (< 25%; Table 1). Five of these missense variants were detected in patients in homozygous form: c.844C > T; p.Arg282Cys (patient 3), (c.139G > A; p.Glu47Lys (patient 16); c.278G > A; p.Gly93Asp (patient 17); c.430G > C; p.Glu144Gln (patient 8), and c.593G > A; p.Arg198His (patient 18). The first four of these five patients presented with a rapidly progressing, severe phenotype associated with early death before 1 year of age (Table 1). The fifth patient (patient 18), homozygous for c.593G > A; p.Arg198His that had an early onset with severe clinical signs, was completely care dependent but was alive at the age of 5 years (Smith et al. 2016). Three of the eight group 1 patients with missense variants were compound heterozygous: c18_24dup; p.Ala9Profs*82 and c134C > T; p.Pro45Leu (sibling patients 6 and 23); c.389G > A; p.Gly130Asp and c.845G > A; p.Arg282His (patient 22). The latter (also described by Edvardson et al. 2013) presented with a severe phenotype as well, being the first CIC patient described with agenesis of corpus callosum and optic nerve hypoplasia. The four patients (nos. 7, 9, 13, and 14) with truncating mutations also had a severe phenotype and died before 1 year of age. Patients 2 and 26 (sibling of patient 3) should also be included in group 1, as they were homozygous for the severe c.844C > G; p.Arg282Gly and c.844C > T; p.Arg282Cys missense mutation, respectively. However, no clinical data could be obtained to confirm this.

The four patients of the second group of SLC25A1-deficiency (nos. 5, 19, 21, and 12) were all compound heterozygous for a severe and a mild allele. Patient 5 was compound heterozygous for missense substitution c.499G > A; p.Gly167Arg (49% activity) and the p.Ala9Profs*82 mutation, which could explain the relatively severe phenotype of the patient, which was associated with death before 2 years of age. Patient 19 was compound heterozygous for a novel frame shift mutation, c.648_655del; p.Met218Serfs*25 and the c.119 T > A; p.Ile40Asn (34% activity). Despite the poor clinical condition of a persistent vegetative state and being dependent on mechanical ventilation and tube feeding, patient 19 was alive (23 months) at the time of the questionnaire; citrate treatment was started at 3 months of age at 800 mg/kg per day, and since then, fewer cerebral attacks were registered. The c.844C > T; p.Arg282Cys missense variant (5% residual activity) was not only detected in the homozygous form in patient 3 (patient group 1) but also in patient 21, compound heterozygous with the c.605 T > C; p.Met202Thr variant (66% activity). The latter was also heterozygous in patient 10 of group 3. Patient 21 had a milder phenotype than patient 3, probably explained by the fact that the second allele of patient 21 harbored a missense variant with high residual CIC activity. Interestingly, this patient also presented clinical features of a myasthenia crisis during respiratory arrest events, precipitated by intercurrent illness. Supplementation with citrate at 800 mg/kg per day prevented any further need for hospitalization during febrile illnesses. The last patient in this group, patient 12, was compound heterozygous for a frameshift mutation and the c.821C > T; r.[821c > t, 820_821del] mutation, involving a splice site. This splice mutation, which mainly results in expression of the frameshift transcript (r. 820_821del; p.Ala274Ilefs*24), may also result in some authentic spliced transcript containing a missense variant (p.Ala274Val; Nota et al. 2013, unpublished data). To investigate the activity of this potential minor transcript r.821c > t (p.Ala274Val), we studied this variant as well, which had 85% residual activity. Patient 12 is the first patient for which the effects of citrate treatment on the clinical course are described in detail (Mühlhausen et al. 2014).

The third group of D/L-2-HGA patients included six cases (five unrelated families), of which four were homozygous (nos. 1, 15, 24, and 25) and two compound heterozygous (nos. 10 and 20). We detected six missense variants with residual activities ranging from 30 to 71% of the activity of wild-type transfectants. The c.578C > G; p.Ser193Trp (31% activity) was detected in two unrelated patients: in homozygous form in patient 1 and heterozygous form in patient 20. Patient 1 was 21 months old at the date of receiving the completed questionnaire, and had early symptom onset at 10 days of age. The patient underwent intermittent respiratory support via continuous positive airway pressure (CPAP) through tracheostomy. Patient 20, aged 3 years, was compound heterozygous for the c.578C > G; p.Ser193Trp and the c.82G > A; p.Ala28Thr variant (71% activity). By comparison, the phenotype of patient 1, homozygous for the c.578C > G; p.Ser193Trp variant, was more severe than that of patient 20. Based on this observation, we speculate that compound heterozygosity of this variant with a higher residual activity missense variant represents an advantage for clinical course and severity of the disease. A similar correlation was observed for patient 10, who was compound heterozygous for the c.605 T > C; p.Met202Thr (66% activity) and c.890A > G; p.Tyr297Cys (30% activity); this patient died at the age of 11 years with typical manifestations of D/L-2HGA. Patients 10 and 20 had a milder clinical phenotypes and were not dependent on mechanical ventilation; tube feeding was only occasionally needed. For patient 20, citrate treatment was initiated at 9 months of age at a dosage of 1500 mg/kg per day and resulted in developmental gains, improved muscle tone, and absence of clinical seizures and apneic events. The effect of citrate treatment in patient 10 cannot be properly assessed because of the very late start in the disease course (at 10.5 years of age) and the very short duration of the trial (1 or 2 weeks, respectively). The homozygous variant, c.740G > A; p.Arg247Gln (52% activity), was present in an adult sibling pair (patients 24 and 25) with a very mild, atypical phenotype, suggestive of congenital myasthenia syndrome (Chaouch et al. 2014). Patient 15 was homozygous for c.784 T > C; p.Cys262Arg variant, which displays 48% residual activity. Intriguingly, despite the relatively high residual activity, this patient presented a severe clinical phenotype of CIC deficiency, with abnormal brain MRI findings and recently developed clinical seizures. This patient (2 years of age) was dependent on intensive medical support. However, he was able to have breathing sprints off the ventilator for 8 h per day, during which time his pulse oximetry remained > 93% on room air. Otherwise, he was ventilator-dependent without the need of supplemental oxygen. In addition to the patients discussed above, two apparently unrelated patients (nos. 4 and 11) were included in this group. In both, the above described c.821C > T mutation (r.[821c > t, 820_821del]) was detected in homozygous form.

### Location of missense mutations in the CIC structural homology model

**Fig. 3:**
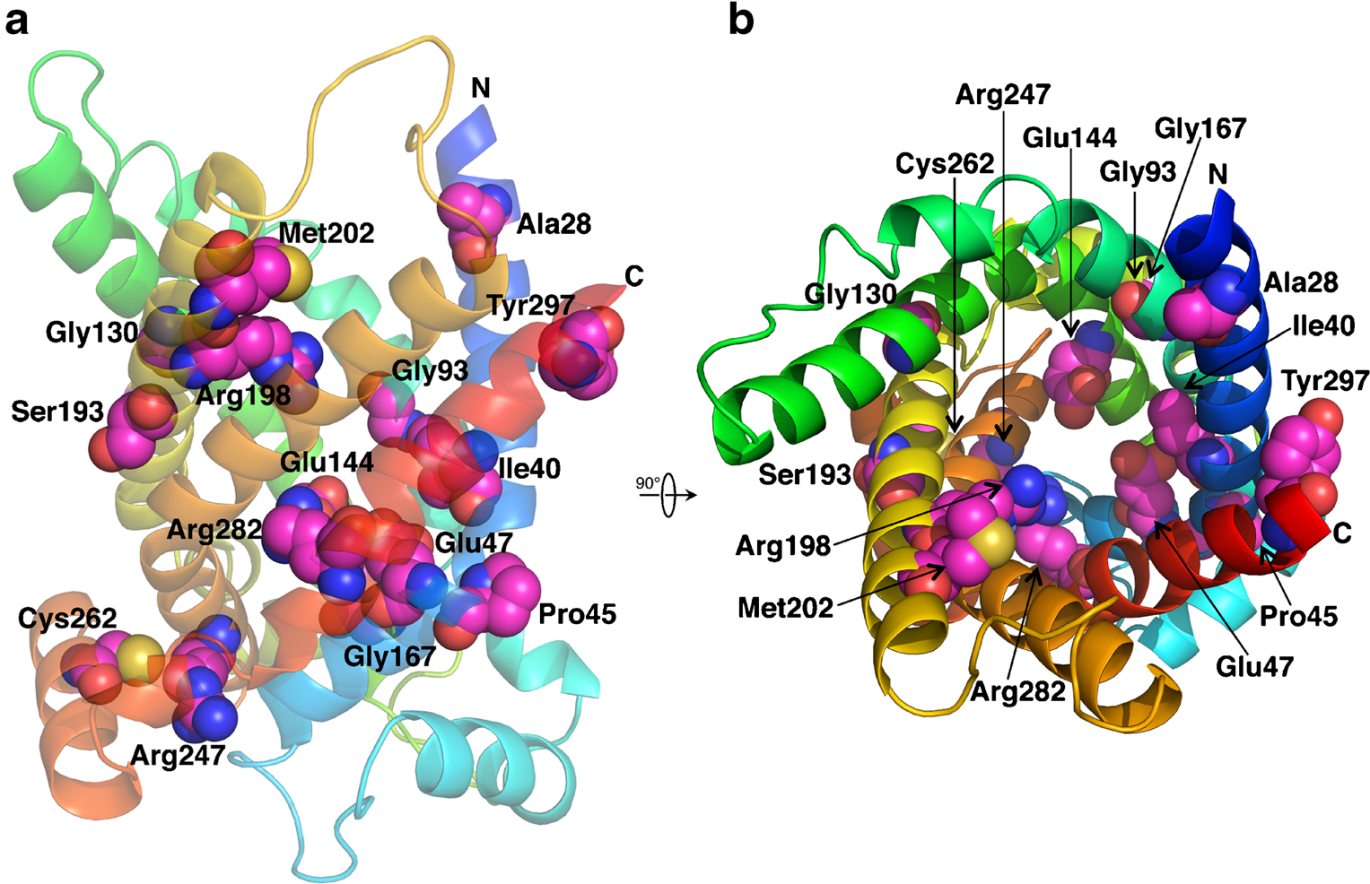
Homology model of human mitochondrial citrate carrier (CIC). The model is displayed with rainbow colors from the N-terminus (*N* blue) to the C-terminus (*C* red). Mutated residues are shown as* spheres*, with carbons in* magenta*. **a **Carrier from the lateral side (in the membrane plane) with the intermembrane space side on the* top* and the mitochondrial matrix side on the* bottom*. **b** Cavity of the carrier as viewed from the intermembrane space side

Positions of missense mutations were mapped onto the homology model of CIC (Fig. 3). It is noteworthy that all residues affected by these missense substitutions have a TS above the threshold of 3.79 (Supplementary Table 2), with the exception of alanine 28, with a TS of 3.40; it also has the highest residual activity of all the disease-causing CIC mutations (Table 1). This observation, together with the fact that various inactivating mutations have been reported at corresponding sites in other MC (Abrams et al. 2015; Ma et al. 2007; Palmieri 2014; Shamseldin et al. 2016; Thompson et al. 2016); (Supplementary Table 2), indicates that these mutations affect residues important in the common structural and functional features of MCF and potentially lead to impaired transport activity.

The single mutations of patients in group 1 are found in functionally important locations within the CIC protein: (1) in the area of the substrate-binding site, consisting of residues in the contact points on each of the three even-numbered transmembrane helices (Robinson and Kunji 2006) and residues in the vicinity of the contact points that also contribute to determine substrate specificity (Marobbio et al. 2008; Monné et al. 2012); (2) in the 3-fold repeated and highly conserved signature motif sequence PX[D/E]XX[K/R]X[K/R]X20–30[D/E]GXXXX[W/Y/F][K/R]G (PROSITE PS50920, PFAM PF00153 and IPR00193), found in members of the mitochondrial carrier family (Palmieri 1994). Here, prolines and first glycines are located in the odd- and even-numbered transmembrane helices, respectively, on the matrix side of the substrate-binding site, constituting the so-called proline–glycine (PG) level 2, implicated in opening and closing the matrix gate of the central substrate translocation pore (Palmieri and Pierri 2010a, b); (3) at PG level 1, where prolines and glycines are found in even- and odd-numbered transmembrane helices on the intermembrane space side, and are suggested to participate in opening and closing the cytoplasmic gate of the central substrate translocation pore (Palmieri and Pierri 2010a, b). It is also noteworthy that all residues affected by these missense substitutions are located in positions with high RS. All RSs of group 1 patients ranging from 5.04 (Glu144Gln) to 6.07 (Arg198His) are above the threshold of 4.68, indicating that these mutations affect residues important in the CIC (Table 2), in keeping with the observed functional data.

**Table 2:** Correlation between average activity, missense alleles, residue-specific scores, and clinical severity

Patient ID	Missense allele 1	Activity (%)	Other allele	Average activity (%)	Residue-specific score^a^	Clinical severity^b^	Patient group
8	Glu144Gln	1	homozygous	1	5.04	severe	1
3 (26)	Arg282Cys	5	homozygous	5	5.61	severe (no data)	1
2	Arg282Gly	7	homozygous	7	5.61	no data	1
17	Gly93Asp	7	homozygous	7	5.5	severe	1
16	Glu47Lys	8	homozygous	8	5.27	severe	1
22	Arg282His	1	Gly130Asp	8.5	5.61/5.50	severe	1
22	Gly130Asp	16	Arg282His	8.5	5.50/5.61	severe	1
18	Arg198His	11	homozygous	11	6.07	severe	1
6 (23)	Pro45Leu	23	frame shift	11.5	5.0/fs	severe (severe)	1
19	**Ile40Asn**	34	frame shift	17	**3.50**/fs	severe	2
5	Gly167Arg	49	frame shift	24.5	5.10/fs	severe	2
1	Ser193Trp	31	homozygous	31	5.51	mild	3
21	Arg282Cys	5	Met202Thr	35.5	5.61/5.33	mild	2
21	Met202Thr	66	Arg282Cys	35.5	5.33/5.61	mild	2
10	Tyr297Cys	30	Met202Thr	48	4.89/5.33	mild	3
15	Cys262Arg	48	homozygous	48	5.51	severe, but intermittent ventilation	3
20	**Ala28Thr**	71	Ser193Trp	51	**3.34**/5.51	mild	3
24 (25)	**Arg247Gln**	52	homozygous	52	**3.38**	mild	3

^a^Residue-specific scores (RS) are a measure of the strength of the evolutionary selection acting on the carrier residues and, hence, of their function and structure relevance. RS scores > 4.68 indicate that the affected residues are considered functionally relevant and/or structurally important. Lower scores are only observed for mutant proteins with considerable residual activity, indicated in bold (p.Ala28Thr, p.Arg247Gln and p.Ile40Asn)

^b^Mild phenotype compared with the severe combined D/L-2-hydroxyglutaric aciduria (HGA) cases

All residues affected by missense substitutions of groups 2 and 3 are found within the CIC protein: (1) inside the carrier cavity but not in the substrate binding area, with one exception; i.e., isoleucine 40; (2) outside the substrate translocation pore toward the membrane and at PG level 1; (3) on the outside of the matrix gate. They all have RSs above the threshold of 4.68, except for alanine 28, with the lowest RS (3.34), which corresponds to the highest residual activity of all disease-associated CIC mutations (71% of WT); arginine 247 with a RS of 3.38, and isoleucine 40 with a RS of 3.50. The latter residues display substantial residual transport activity (52% and 34%, respectively) (Table 2).

## Discussion

We present an overview of the clinical phenotypes of 26 genetically diagnosed D/L-2-HGA patients, including eight novel cases. To make a first prediction for clinical outcome, we classified patients into three groups based on their phenotype and genotype, as described in the “Results” section: (1) two severe alleles (with < 25% residual activity), (2) one severe and one mild allele, and (3) two mild alleles. The importance of mutated residues is evaluated using the scoring system developed by Pierri et al. and by the newly developed functional studies.

A detailed discussion on the different mutations and their activities and classification in the three groups is provided in the supplementary data (Palmieri et al. 2011; Ruprecht et al. 2014; von Heijne 1996).

This study provides a first observation that a genotype–phenotype correlation exists in this small cohort of patients with D/L-2HGA. Lack of residual SLC25A1 transporter activity (severe missense mutation or truncating mutation) is likely to contribute to a severe disease presentation associated with early death. We also noted a strong positive correlation between extensive medical care and increased life expectancy, irrespective of type of mutation/residual activity. This may partly be explained by altering the natural history of the disease by preventing apneic episodes, aspiration risk, and respiratory arrest by ventilation support and improving metabolic status by improved nutrition through g-tube feeding. It is of interest to note that there is an alternative route for citrate production in the cytoplasm by reductive carboxylation (Jiang et al. 2016), which may explain the presence of some residual citrate levels. However, this is apparently not sufficient to prevent illness. Identifying functionally important residue positions in MC by RS, introduced by Pierri et al. 2014, has proven to be very valuable in classifying missense mutations in CIC (Table 2). All mutated residues with RSs below the threshold value correspond to mutant proteins with considerable residual activity (p.Ala28Thr, p.Arg247Gln and p.Ile40Asn). However, there is no direct relationship between RS above the threshold and the level of transport activity of a mutated protein, because their activity not only depends on the structural/functional importance of the original residue but also on the kind of substitution: changes in side-chain size, charge, etc. Nevertheless, RSs > 5.6 are associated with mutant proteins with a very low residual activity (≤11% of wild type). 

It should be noted that our study has some limitations. First, our functional assay is a steady-state measurement of [^13^C_2_] citrate accumulation in extracellular medium after prolonged culture with [U-^13^C_6_] glucose and is an indirect activity measurement, most likely overestimated, and proxy of actual citrate transport activity of a single allele across the inner mitochondrial membrane *in vivo*. Second, our assay cannot simultaneously measure the activity of two alleles, meaning that it does not allow us to mimic compound heterozygous patients, where the degree of CIC functional deficiency is the result of the contribution of both alleles. Although it is not known whether increased expression levels of both or one allele in cells of compound heterozygous patients might in part compensate for low-activity mutant proteins, activity in these cells can be estimated by averaging the activity levels measured separately for both alleles. By doing so, we observed that in the case of compound heterozygosity also, an *in vitro* activity < 25% of wild-type transfectants can be associated with a more severe phenotype (Table 2). 

In conclusion, our newly developed functional assay can be used, together with structural data and residue-specific scores, as an assisting tool for interpreting new missense variants and may be of added value for physicians in counseling parents of patients with (missense) variants in CIC.

## Electronic supplementary material

Supplementary Table 1 (DOCX 33 kb)

Supplementary Table 2 (DOCX 39 kb)

Supplementary Fig. 1 Western blot analysis of mitochondrial citrate carrier (CIC) alleles containing missense variants transfected into primary *SLC25A1* ^−/−^-deficient fibroblasts to confirm construct validity. A representative Western blot analysis of triplicate experiments is shown. CIC relative abundance was analyzed by Western blotting using antibodies against SLC25A1 protein and actin. Although these blots are not quantitative, apparent reduced CIC accumulation of alleles containing certain missense variants is observed, which is probably explained by instability of the CIC transcript or protein due to the presence of the missense mutation. However, it cannot be excluded that minor differences in transfection efficiency may have partly contributed to these variations. The p.Tyr256* allele was transfected as a negative control. (GIF 75 kb)

High-resolution image (TIFF 2071 kb)

ESM 1 (DOCX 62 kb)
