# Supplementary material for: An overview of combined D-2- and L-2-hydroxyglutaric aciduria: functional analysis of CIC variants
Source: J Inherit Metab Dis. 2017 Dec 13;41(2):169–80. doi: 10.1007/s10545-017-0106-7 (PMC5830478; doi:10.1007/s10545-017-0106-7)
Supplement: Supplementary file 1 — (DOCX 33 kb) [file 10545_2017_106_MOESM1_ESM.docx]

**Supplementary Table 1. Urinary levels of Krebs cycles metabolites in patients with D/L-2-HGA.**

| Patient | Citrate | Isocitrate | D-2-HG | L-2-HG | Malate | Fumarate | Succinate | | α-KG | |
| --- | --- | --- | --- | --- | --- | --- | --- | --- | --- | --- |
| 13 | 718 | 109 | 583 | 226 | 800 | 174 | 891 | 5139 | |  |
| 14 | 199 | 59 | 484 | 146 | 253 | 44 | 170 | 1981 | |  |
| 15 | 832 | 103 | 351 | 90 | 737 | 123 | 646 | 1074 | |  |
| 16 | 825 | 179 | 610 | 184 | 1154 | 243 | 389 | 7321 | |  |
| 17 | 91 | 68 | 286 | 94 | 120 | 14 | 149 | 1908 | |  |
| 19 | 311 | 66 | 542 | 145 | 427 | 79 | 176 | 4396 | |  |
| 20 | 1157 | 91 | 81 | 27 | 115 | 15 | 220 | - | |  |
|  |  |  |  |  |  |  |  |  | |  |
| control range | 55-2756 | 23-226 | 2.8-17 | 1.3-19 | 0.4-17.8 | 0.3-6.1 | 12.2-306 | 29.8-117 | |  |

Citrate, isocitrate, D-2-hydroxyglutarate (D-2-HG), L-2-hydroxyglutarate (L-2-HG), malate, fumarate, succinate and α-ketoglutarate (α-KG) levels (mmol/mol creatinine) in urine samples of individuals with combined D/L-2-HGA. Urinary citrate, isocitrate, succinate, α-KG, fumarate and malate were measured by liquid chromatography-tandem mass spectrometry (LC-MS/MS) (Blom et al 2007) with some modifications. Briefly, to 5 µL of urine a mixture of 0.4 nmol ^2^H_4_-citrate, 0.4 nmol ^13^C_4_-fumarate, 0.4 nmol ^13^C_4_-succinate, 0.8 nmol ^2^H_3_-L-malate and 0.4 nmol ^2^H_4_- α-ketoglutarate was added as internal standard. Samples were mixed and 6 µL of the sample was injected onto the LC-MS/MS. For the quantification of citrate and isocitrate, the following multiple reaction monitoring transitions were used: citrate m/z -191 > -87, ^2^H_4_-citrate m/z -195 > -89, isocitrate m/z -191 > -73, succinate m/z -117 > 73, ^13^C_4_-succinate m/z -121 > 76, fumarate m/z -115 > 71, ^13^C_4_-fumarate m/z -119 > 74, malate m/z -133 > -115, ^2^H_3_-L-malate m/z -136 > 117, α-ketoglutarate m/z -145 > 57 and ^2^H_4_- α-ketoglutarate m/z -149 > 60. D-2-HG and L-2-HG were measured by LC-MS/MS according to (Struys et al 2004). Control ranges are taken from (Nota et al 2013).
