# Supplementary material for: An overview of combined D-2- and L-2-hydroxyglutaric aciduria: functional analysis of CIC variants
Source: J Inherit Metab Dis. 2017 Dec 13;41(2):169–80. doi: 10.1007/s10545-017-0106-7 (PMC5830478; doi:10.1007/s10545-017-0106-7)
Supplement: Supplementary file 2 — (DOCX 39 kb) [file 10545_2017_106_MOESM2_ESM.docx]

**Supplementary Table 2: Position of the 17 missense variants in the CIC homology model and their predicted and observed importance for structure and function.**

| Mutation in human CIC protein | Position of the mutated residues in the primary structure ^1^ | Position of the mutated residues in the 3D homology model ^2^ | Transversal scores of the mutated residues ^3^ | Inactivation- or disease-causing mutations in other mitochondrial carriers ^4^ |
| --- | --- | --- | --- | --- |
| p.Ala28Thr | H1 | cavity | 3.40 | - |
| p.Ile40Asn | H1 | cavity, binding site | 3.94 | ScAac2p: K38A  ScMir1p: H32A |
| p.Pro45Leu | H1, signature motif | external, PG level 2 | 4.71 | bOGC: P41C |
| p.Glu47Lys | H1, signature motif | cavity, matrix gate | 4.27 | ScMir1p: D39N  ScCtp1p: E34C ^5^  bOGC: D43C  SLC25A20: D32N  SLC25A13: D350N |
| p.Gly93Asp | H2 | cavity, binding site | 4.46 | ScAac2p: R96A  bOGC: R90C  HamsterSLC25A32: G91L  SLC25A4: R80H ^6^  SLC25A20: G81R |
| p.Gly130Asp | H3 | external, PG level 1 | 3.84 |  |
| p.Glu144Gln | H3, signature motif | cavity, matrix gate | 4.74 | ScAac2p: D149S  ScMir1p: E137Q  ScCtp1p: E131C ^5^  bOGC: E141C  SLC25A20: E132A  HamsterSLC25A32: W142A |
| p.Gly167Arg | h34 | external | 4.07 | - |
| p.Ser193Trp | H4 | external, PG level 1 | 4.37 | SLC25A38: P190R |
| p.Arg198His | H4 | cavity, binding site | 4.31 | ScCtp1p: R189C  ScMir1p: K187A  bOGC: Q198C |
| p.Met202Thr | H4 | cavity, aromatic belt | 4.05 | bOGC: Y202C |
| p.Arg247Gln | H5, signature motif | external, matrix-gate | 4.42 | ScAac2p: R254I  bOGC: R246C  SLC25A46: R340C ^7^ |
| p.Cys262Arg | h56 | external | 3.84 | - |
|  |  |  |  |  |
| p.Arg282Gly | H6 | cavity, binding site | 4.10 | ScCtp1p: R276C  ScMir1p: R276A  SLC25A13: R585H  SLC25A15: T272I  SLC25A38: R278G  SLC25A42: N291D ^8^ |
| p.Arg282Cys |  |  |  |  |
| p.Arg282His |  |  |  |  |
| p.Tyr297Cys | H6 | external, aromatic belt | 4.01 | HamsterSLC25A32:  Y300A |

^1^) “H” stands for transmembrane helices; “h” stands for matrix short helices (Palmieri 2013; Palmieri 2014). ^2^) “cavity” refer to amino acids whose side chains protrude in the carrier’s cavity; “external” refer to the ones that do not. The “cavity” is the space surrounded by H1-H6 in the carrier conformation state, called “c-state”, and is believed to be part of the path through which the substrate (in this case citrate or malate) is translocated from one side of the membrane to the other (Pierri et al 2014) (Figure 3). ^3^) “Transversal scores” are a measure of the strength of the evolutionary selection acting on the carrier residues and, hence, of their function and structure relevance. A residue with a TS higher than 3.79 is above the median value and considered functionally and/or structurally important in the common MCF structure and transport mechanism; ^4^) “Inactivation- (less than 10% of wt activity) or disease-causing mutations” found in other mitochondrial carriers at corresponding sites of the 17 mutations discovered in CIC, (Pierri et al 2014). ^5^) (Ma et al 2007). ^6^) (Thompson et al 2016). ^7^) (Abrams et al 2015). ^8^) (Shamseldin et al 2016).
