# Supplementary figures and images for: An overview of combined D-2- and L-2-hydroxyglutaric aciduria: functional analysis of CIC variants
Source: J Inherit Metab Dis. 2017 Dec 13;41(2):169–80. doi: 10.1007/s10545-017-0106-7 (PMC5830478; doi:10.1007/s10545-017-0106-7)

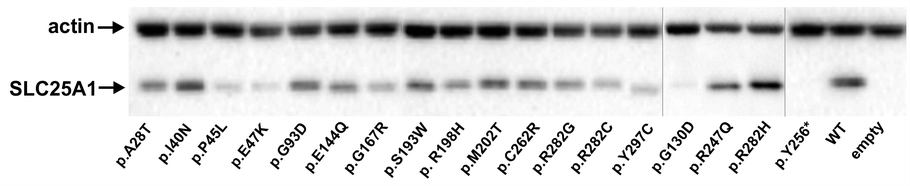

Supplement: Supplementary file 3 — Western blot analysis of mitochondrial citrate carrier (CIC) alleles containing missense variants transfected into primary SLC25A1 −/−-deficient fibroblasts to confirm construct validity. A representative Western blot analysis of triplicate experiments is shown. CIC relative abundance was analyzed by Western blotting using antibodies against SLC25A1 protein and actin. Although these blots are not quantitative, apparent reduced CIC accumulation of alleles containing certain missense variants is observed, which is probably explained by instability of the CIC transcript or protein due to the presence of the missense mutation. However, it cannot be excluded that minor differences in transfection efficiency may have partly contributed to these variations. The p.Tyr256* allele was transfected as a negative control. (GIF 75 kb) [file 10545_2017_106_Fig4_ESM.gif]
