# Supplementary material for: An overview of combined D-2- and L-2-hydroxyglutaric aciduria: functional analysis of CIC variants
Source: J Inherit Metab Dis. 2017 Dec 13;41(2):169–80. doi: 10.1007/s10545-017-0106-7 (PMC5830478; doi:10.1007/s10545-017-0106-7)
Supplement: Supplementary file 5 — (DOCX 62 kb) [file 10545_2017_106_MOESM4_ESM.docx]

**Supplementary data: In depth analysis of CIC proteins containing missense mutations.**

The c.134C>T; p.Pro45Leu, found in the first group, displays the highest residual activity (23%). Proline 45 is part of PG level 2 and the first repeat of the signature motif sequence. The prolines of the signature motif sequence kink the odd-numbered transmembrane helices in the "cytosolic" conformation of the carrier demonstrated by X-ray crystallography (Pebay-Peyroula et al 2003) and they are implicated in the conformational changes of the catalytic transport cycle (Pebay-Peyroula et al 2003; Palmieri and Pierri 2010; Ruprecht et al 2014). The fact that proline at this position is conserved in all 53 human mitochondrial carriers, its important structural role and the RS of this site above the gene threshold (5.00), strongly supports that this missense substitution affects citrate transport rate. This variant is found in heterozygous form in 2 siblings, patient 6 and 23 (Nota et al 2013; Prasun et al 2015). The compound heterozygosity with the truncating mutation p.Ala9Profs*82, could explain the very severe phenotype of these patients, associated with early death before the age of 2 months. Glutamates 47 and 144 (together with aspartate 242) are the negatively charged residues of the first portion of the signature motif sequence and take part in the formation of a salt bridge network inside the substrate translocation pore that contributes to the formation of the matrix gate (Pebay-Peyroula et al 2003; Palmieri and Pierri 2010; Ruprecht et al 2014). Therefore, p.Glu47Lys and p.Glu144Gln mutants lead to the loss of the negative charges implicated in the substrate transport mechanism (Palmieri and Pierri 2010; Palmieri et al 2011; Palmieri 2013; Ruprecht et al 2014). The RS of 5.27 and 5.04 of these positions, are in agreement with the detrimental effects on CIC activity in the homozygous patients 8 and 16. Overexpression of p.Gly93Asp, p.Arg198His, p.Arg282Gly, p.Arg282Cys and p.Arg282His exhibits residual citrate transport activity below 11%. Glycine 93, arginine 198 and arginine 282 are located in the substrate binding area on transmembrane helix 2, 4 and 6, respectively: glycine 93 is part of contact point I whereas the arginines 198 and 282 protrude in the carrier cavity one helix turn above contact points II and below contact point III, respectively. The diminished activities of p.Gly93Asp, p.Arg198His, p.Arg282Gly, p.Arg282Cys and p.Arg282His are most likely caused by alterations of substrate binding. The positional RS of 5.50, 6.07 and 5.61 for glycine 93, arginine 198 and arginine 282, respectively, are in line with the severe clinical features of the homozygous patients 17,18 and 3 and contribute to the clinical phenotype of the compound heterozygous patient 22. Pathogenicity caused by mutations of arginines 198 (c.593G>A; p.Arg198His) and 282 (c.844C>G; p.Arg282Gly, c.844C>T; p.Arg282Cys and c.845G>A; p.Arg282His) has been established previously (Edvardson et al 2013; Nota et al 2013; Smith et al 2016). Primary deficient fibroblasts of homozygous patients with p.Arg282Gly and p.Arg282Cys grown in [U-^13^C_6_] glucose enriched medium showed about 12 and 6% [^13^C_2_] citrate levels, respectively, in the culture medium compared to controls, demonstrating that these specific mutations result in impaired citrate efflux (Nota et al 2013). In addition, overexpressed and reconstituted recombinant yeast CIC with the mutation p.Arg276Cys (which corresponds to the human p.Arg282Cys) displayed diminished transport activity (Ma et al 2007). Furthermore, the yeast CIC mutant corresponding to human CIC p.Arg282His has virtually no citrate transport activity as assessed by recombinant expression and direct transport assays into reconstituted liposomes (Edvardson et al 2013). All these findings are in support of the similar residual transport activities of p.Arg282Gly, p.Arg282Cys and p.Arg282His reported here. The last mutant of patients group 1 c.389G>A; p.Gly130Asp, which has 16% residual activity in our experimental setup, is found at GP level 1 in transmembrane helix 3 facing the lipid bilayer. The introduction of an aspartate in the hydrophobic environment of this position might therefore have strong deleterious effects on the protein-lipid contacts and cause local perturbations in the secondary structure that could affect the conformational changes of the transporter involved in substrate translocation. The substitution c.389G>A; p.Gly130Asp was previously assessed by the corresponding mutation in yeast CIC by direct transport assays into reconstituted liposomes showing 25% residual activity (Edvardson et al 2013). The discrepancy between these two measurements could be ascribed to the different conditions of the two assay systems (initial transport rates being measured by Edvardson et al) and in this particular case to the lipid composition. The low residual activity of p.Gly130Asp, its position towards the hydrophobic core of the membrane and a RS of 5.50 suggests a contribution of this mutation to the phenotype of the compound heterozygous patient 22 (p.Arg282His/p.Gly130Asp).

The CIC mutations that are classified as mild alleles (higher than 25% residual activity) are discussed below. These are p.Ala28Thr, p.Ile40Asn and p.Met202Thr which display 71, 34 and 66% residual activity. The first two residues are located in transmembrane helix 1 and the third in transmembrane helix 4 and they all protrude into the central substrate translocation pore with their side chains but none of them are found in the signature motifs or implicated in substrate binding. Whereas alanine 28 and methionine 202 are towards the intermembrane space side of the carrier, isoleucine 40 is close to the level thought to constitute the substrate binding site, which might explain the larger impact on transport activity of the mutation in this position. However, it is worth noting that none of these substitutions involve large changes in the side chains in terms of size and charge compared to many of the other mutations in CIC. These considerations and the RS of 3.34 for alanine 28 and of 3.50 for isoleucine 40, might explain, at least in part, the somewhat milder phenotypes in the compound homozygous patients 10, 20 and 21, compared to those of group 1 patients. The overexpression assay showed that p.Ser193Trp and p.Tyr297Cys have 31 and 30% residual activity, respectively. Serine 193 is situated in transmembrane helix 1 at PG level 1 with its side chain exposed towards the hydrophobic core of the membrane and has a RS of 5.51. Therefore, this mutation probably has an effect on the carrier conformational changes occurring in the transport mechanism and lipid contacts rather than on substrate binding. A previous study of primary deficient fibroblasts derived from patient 1, also confirmed the deleterious effect of this mutation that was shown to have about 13% residual activity (Nota et al 2013). Tyrosine 297 is located outside the substrate translocation pore on the intermembrane space side of transmembrane helix 6 in the aromatic belt (Pierri et al 2014), which corresponds to aromatic residues that are positioned in the lipid head group region of the membrane in many membrane proteins (von Heijne 1996). This residue position has a RS of 4.89, close to the gene-specific threshold of 4.68. These considerations lie beneath the clinical features found in compound heterozygous patients 10 and 20. The mutations p.Gly167Arg, p.Arg247Gln and p.Cys262Arg have residual activities of 49, 52 and 48%, respectively. The activity of primary fibroblasts with p.Gly167Arg has been previously estimated to be about 20% (Nota et al 2013). This result is in good agreement with the residual activity of p.Gly167Arg in the overexpression studies described here as the other allele has a frame-shift mutation leading to a truncated, presumably inactive form of CIC. The yeast CIC mutant corresponding to human p.Arg247Gln resulted to have about 40% of wild type activity in the reconstituted system (Chaouch et al 2014) which is similar to the results obtained here. The mutations p.Gly167Arg, p.Arg247Gln and p.Cys262Arg are found in the matrix gate area of the CIC homology model outside the central cavity. Glycine 167 and cysteine 262 are found in the highly variable sequences forming the matrix helices h34 and h56, respectively, whereas arginine 247 is the second positively charged residue of the third signature motif forming a salt bridge with the second negatively charged residue of the same signature motif in the cytosolic conformation of the carrier (Pebay-Peyroula et al 2003; Ruprecht et al 2014). Glycine 167, arginine 247 and cysteine 262 have the RSs 5.10, 3.38 and 5.51, respectively, and the mutations of these residues, could potentially have effects on matrix gate opening and closing. This rational is suggested to form the molecular basis for compound heterozygous patient 5 and the homozygous patients 15, 24 and 25.

The last interesting variant to discuss is r.[821c>t, 820_821del] splice mutation. Primary fibroblasts of a patient homozygous for this mutation (patient 4) showed 57% citrate efflux, and at the protein level, some CIC protein was detected on Western blot (Nota et al 2013). The presence of this protein could be either explained by some authentic spliced protein product containing a p.Ala274Val missense variant, and/or alternatively, because the frameshift occurs at the C-terminal end resulting in the replacement of 37 amino acids by 24 others (Nota et al 2013). Therefore, the activity of the authentic splice site, which gives rise to the Ala274Val, was studied and showed 85% residual activity. Thus, potentially both transcripts (the one lacking the 37 authentic C-term amino acids, or the missense variant) may have some residual activity. This is in line with the fact that patients carrying this mutation usually present with a relatively longer life expectancy (Table 1).
